# Phase I study of glofitamab in Japanese patients with relapsed or refractory B-cell non-Hodgkin lymphoma

**DOI:** 10.1007/s10147-026-03110-2

**Published:** 2026-07-02

**Authors:** Yuko Shirouchi, Yuko Mishima, Suguru Fukuhara, Shinichi Makita, Koji Izutsu, Yasuhito Terui, Takuro Suzuki, Atsuko Kawasaki, Takeshi Miyake, Dai Maruyama

**Affiliations:** 1 Department of Hematology Oncology, Cancer Institute Hospital, Japanese Foundation for Cancer Research, Tokyo, Japan; 2 Department of Hematology, National Cancer Center Hospital, Tokyo, Japan; 3 Department of Hematology, Saitama Medical University Hospital, Saitama, Japan; 4 Chugai Pharmaceutical Co., Ltd, Tokyo, Japan

**Keywords:** Glofitamab, Bispecific antibody, Relapsed/refractory B-cell lymphoma

## Abstract

**Background:**

Glofitamab is a T-cell engaging CD20xCD3 bispecific antibody approved for treatment of relapsed/refractory diffuse large B-cell lymphoma (DLBCL) in the US and Europe.

**Methods:**

This phase I study (jRCT2080225077) investigated safety, pharmacokinetics, and efficacy of glofitamab in Japanese patients with relapsed/refractory B-cell non-Hodgkin lymphoma (B-NHL). Patients received obinutuzumab pretreatment (1000 mg) 7 days before first glofitamab administration. Glofitamab was given with step-up dosing on Cycle (C)1 Day (D)1 and D8, then at target dose every 3 weeks from C2D1 (7 days after C1D8) (Cohort 1: 2.5/10/16 mg; Cohort 2: 2.5/10/30 mg) until progression. Primary endpoints were safety and pharmacokinetics; secondary endpoint was efficacy.

**Results:**

Eight patients with B-NHL (DLBCL, *n* = 5; follicular lymphoma [FL], *n* = 2; transformed FL, *n* = 1) were enrolled (Cohort 1, *n* = 5; Cohort 2, *n* = 3; median age, 64.5 years; Ann Arbor Stage III/IV, *n* = 7). The two patients with FL received no glofitamab due to hematological adverse events (AEs) after obinutuzumab. Median number of glofitamab cycles was 48 (Cohort 1) and 43 (Cohort 2). Seven patients had grade 3/4 AEs; no grade 5 AEs were reported. Two patients had serious AEs; no dose-limiting toxicities were observed. Cytokine release syndrome occurred in all glofitamab-treated patients (grade 1/2/3: *n* = 3/*n* = 2/*n* = 1), predominantly in C1. Serum glofitamab concentrations showed similar time profiles in both cohorts, and exposure increased in a dose-proportional manner. Overall and complete response rates were 75.0% (6/8 patients; Cohort 1: 3/5 patients [60.0%]; Cohort 2: 3/3 patients [100%]).

**Conclusion:**

Glofitamab demonstrated a manageable safety profile with encouraging response rates in Japanese patients with relapsed/refractory B-NHL.

**Supplementary Information:**

The online version contains supplementary material available at 10.1007/s10147-026-03110-2.

## Introduction

Since the introduction of the anti-CD20 monoclonal antibody (mAb) rituximab to standard chemotherapy, the mortality rate of B-cell non-Hodgkin lymphoma (B-NHL) has decreased [1]. However, approximately one third of patients will eventually develop relapsed or refractory (R/R) B-NHL [2].

Current standard of care (SoC) for R/R B-NHL includes conventional chemotherapy in combination with anti-CD20 mAbs [3] and autologous stem cell transplant (ASCT) [4]. However, many patients are ineligible for ASCT due to age, comorbidities, refractoriness to chemotherapy, and poor performance status [4, 5]. Currently approved treatment options for R/R B-NHL include chimeric antigen receptor (CAR) T-cell therapies, bispecific antibodies, antibody drug conjugates, and small molecule targeted therapies. CAR T-cell therapies have recently been approved for R/R diffuse large B-cell lymphoma (DLBCL), however they have limitations including logistical challenges such as long manufacturing time and the need for access to authorized CAR T-cell treatment centers with trained staff; safety risks such as cytokine release syndrome (CRS) and neurological adverse events (AEs); and patient ineligibility [6]. The prognosis for patients with R/R B-NHL is poor [5, 7, 8], and therefore new off-the-shelf treatment options which have high efficacy are needed to improve outcomes for these patients.

To date, bispecific mAbs such as glofitamab have shown high response rates as monotherapy, and promising results when combined with chemotherapy in a R/R setting [9, 10]. Glofitamab is a CD20xCD3 bispecific antibody; with its 2:1 tumor-T-cell binding configuration, it confers bivalency for CD20 (B cells) and CD3 (T cells), leading to the engagement and redirection of patients’ existing T cells to eliminate malignant B cells [11, 12]. Glofitamab’s high affinity for CD20, due to its two distinctive CD20 binding sites, is preserved in the presence of competing mAbs, allowing for pre- or co-treatment with other anti-CD20 agents [11]. It is an off-the-shelf medication; hospitalization may be required for receipt of the first dose and it can be administered as monotherapy or in combination with chemotherapy after pretreatment with obinutuzumab [8].

Glofitamab has demonstrated high response rates as monotherapy and as combination therapy in patients with R/R B-NHL [8–10]. In a pivotal phase I/II global study, glofitamab monotherapy was administered intravenously (IV) for 12 cycles, with step-up dosing in C1, to patients with R/R DLBCL who had received ≥ 2 prior therapies. Overall response rate (ORR) and complete response (CR) rate were 52.0% and 39.0%, respectively [8]. At a maximum follow-up of 27.4 months, 84.1% of patients who achieved a CR at end of treatment had an ongoing CR [9]. Glofitamab had a predictable and manageable safety profile, with the use of obinutuzumab pretreatment and glofitamab step-up dosing providing effective CRS mitigation [8]. Based on these results, glofitamab monotherapy has been approved in more than 60 countries worldwide.

Given the encouraging data from the global study [8], a phase I multicenter, open-label, dose-escalation study (jRCT2080225077) of glofitamab as a single agent (post-obinutuzumab pretreatment) in Japanese patients with R/R B-NHL was conducted to assess the safety, tolerability, and pharmacokinetics (PK) of glofitamab. We report the primary results of this trial.

## Methods

### Study design

This was a multicenter, open-label, dose-escalation phase I trial (jRCT2080225077) designed to enroll 6–12 patients across two cohorts. The study included a dose-limiting toxicity (DLT) evaluation period for each patient, starting on cycle (C)1 day (D)1 and ending on C3D1. C1 was 14 days and subsequent cycles were 21 days.

The trial protocol was approved by the institutional review board at each participating center. The trial was conducted in accordance with the principles of the Declaration of Helsinki and the International Conference on Harmonisation Guidelines for Good Clinical Practice. All patients provided written informed consent prior to enrollment.

### Patients

Patients were eligible if they were aged ≥ 20 years, had histologically confirmed CD20-positive B-NHL, and an Eastern Cooperative Oncology Group performance status of 0 or 1. Key exclusion criteria included central nervous system (CNS) lymphoma or other CNS disease, and prior CD20/CD3-targeting therapy. Full details of inclusion and exclusion criteria are provided in the Supplementary Appendix.

### Treatment

Eligible patients were enrolled in two cohorts to receive glofitamab at two different target doses. Glofitamab was administered intravenously (IV) as step-up doses (to mitigate the risk of CRS) on D1 (2.5 mg) and D8 (10 mg) of C1. Seven days after C1D8, glofitamab was then administered at a target dose (Cohort 1: 16 mg; Cohort 2: 30 mg) on D1 of every cycle from C2 onwards. In both cohorts, obinutuzumab pretreatment (1000 mg IV) was given seven days before the first dose of glofitamab to further mitigate CRS.

If an AE was related to obinutuzumab pretreatment, resolution of non-hematological toxicities and thrombocytopenia to grade 1 or less and hematological toxicities (excluding lymphopenia) to grade 2 or less was required before initiation of glofitamab treatment.

Glofitamab administration was interrupted if infusion-related reactions (IRRs) or CRS occurred and resumed when resolved. In case of IRR/CRS, tocilizumab and corticosteroids could be administered at the discretion of the investigator. Further details on CRS management can be found in the Supplementary Appendix. Patients who did not meet discontinuation criteria (see Supplementary Appendix) were able to continue glofitamab treatment until progressive disease (PD) if determined appropriate by the study investigators.

### Endpoints and assessments

Multiple primary endpoints were set for this trial and were as follows: safety, tolerability, and PK of glofitamab monotherapy following obinutuzumab pretreatment in patients with B-NHL, and the DLTs of glofitamab. Secondary endpoints included AEs for obinutuzumab, immunogenicity (anti-drug antibodies [ADAs]) for glofitamab post-obinutuzumab, and efficacy (investigator-assessed ORR, duration of response [DOR], and progression-free survival [PFS]; see Supplementary Appendix for efficacy endpoint definitions)**.**

AEs were graded according to NCI CTCAE v5.0; the timing of AE onset was also assessed. CRS was graded by the ASTCT 2019 criteria (see Supplementary Appendix for further details on CRS management) [13]. Interleukin-6 (IL-6) was centrally assessed using blood or serum.

Response to treatment was investigator-assessed and based on physical examination, computed tomography/magnetic resonance imaging, optional positron emission tomography, and bone marrow biopsy. Response was graded per the Lugano response criteria [14]. An exploratory, post-hoc efficacy analysis was performed in patients treated with glofitamab. PK was assessed using serum concentrations of patients who received at least one dose of obinutuzumab or glofitamab, including parameters of area under the curve (AUC), maximum serum concentration (C_max_), and AUC from time 0 to 35 days after the first glofitamab dose (AUC_0–35_). Immunogenicity was assessed in patients with at least one ADA before and after treatment.

### Statistical analysis

Patients who received at least one dose of obinutuzumab or glofitamab were included in the safety and efficacy analyses. Those who received obinutuzmab pretreatment but did not receive subsequent glofitamab treatment were also included in the efficacy and safety analyses. Patients included in PK analysis are described in the Supplementary Methods.

The sample size for the study was not based on any formal statistical hypothesis. The study was conducted in two cohorts using a 3 + 3 design. If a patient dropped out due to a DLT or other reasons, an additional patient could be enrolled into the cohort. The safety of glofitamab was evaluated by summarizing AEs, laboratory data, vital signs, and electrocardiograms by cohort. PK parameters were presented as mean (± standard deviation [SD]) unless otherwise stated. A Clopper-Pearson estimate was used to provide 95% confidence intervals (CIs) for the ORR.

## Results

### Patients

Between February 2020 and January 2024, eight patients were enrolled (Cohort 1, *n* = 5; Cohort 2, *n* = 3) and included in the safety and efficacy analyses. Five patients had DLBCL, two patients had follicular lymphoma (FL), and one patient had transformed FL. The two patients with FL were enrolled to Cohort 1 but discontinued the study treatment before glofitamab administration and were not included in the exposure analysis. These patients were included in the PK analysis but did not have glofitamab serum concentration measurements, therefore the PK findings for Cohort 1 were substantially driven by the three glofitamab-treated patients. Additionally, the two patients with FL did not enter the DLT evaluation period.

Median age was 64.5 years (range 55–76). Most patients (87.5%, *n* = 7) had advanced disease (Ann Arbor stage III–IV). The median number of prior lines of therapy was four in Cohort 1 and one in Cohort 2; all patients had received a prior anti-CD20 antibody (Table 1). Two patients had bone marrow involvement.

**Table 1:** Demographic and clinical characteristics at baseline

*n* (%) unless stated	Cohort 1 *n* = 5	Cohort 2 *n* = 3	All patients *N* = 8
Median age (range), years	59.0 (55–73)	71.0 (68–76)	64.5 (55–76)
Male	2 (40.0)	1 (33.3)	3 (37.5)
Median bodyweight (range), kg	62.1 (48.0–85.8)	48.5 (40.9–60.9)	59.9 (40.9–85.8)
ECOG PS			
0	5 (100)	3 (100)	8 (100)
1	0	0	0
Ann Arbor stage			
I–II	0	1 (33.3)	1 (12.5)
III–IV	5 (100)	2 (66.7)	7 (87.5)
Histological subtype			
FL	2 (40.0)	0	2 (25.0)
DLBCL	2 (40.0)	3 (100)	5 (62.5)
trFL	1 (20.0)	0	1 (12.5)
Number of prior lines of therapy			
Median, range	4 (2–7)	1 (1–2)	3 (1–7)
1	0	2 (66.7)	2 (25.0)
≥ 2	5 (100)	1 (33.3)	6 (75.0)
Prior anti-lymphoma therapy			
Chemotherapy, anthracycline	5 (100)	2 (66.7)	7 (87.5)
Chemotherapy, non-anthracycline	5 (100)	1 (33.3)	6 (75.0)
Anti-CD20 antibody	5 (100)	3 (100)	8 (100)
CAR T-cell therapy	0	0	0

CAR, chimeric antigen receptor; DLBCL, diffuse large B-cell lymphoma; ECOG PS, Eastern Cooperative Oncology Group performance status; FL, follicular lymphoma; trFL, transformed follicular lymphoma

### Glofitamab exposure

The median (range) number of glofitamab cycles received was 48 (40–56) in Cohort 1 and 43 (42–44) in Cohort 2, excluding the two patients who did not receive glofitamab. The mean (SD) cumulative dose of glofitamab and the median glofitamab dose intensity, respectively, were 764.5 mg (128.0) and 100% in Cohort 1 and 1272.5 mg (30.0) and 100% in Cohort 2.

### Safety

No DLTs were observed in either cohort. In Cohort 1, the two patients with FL who discontinued the study before glofitamab administration did so due to hematological toxicities after obinutuzumab pretreatment. Bone marrow involvement was observed in one of the two patients with FL.

AEs were reported in seven (87.5%) patients after obinutuzumab administration but before glofitamab administration; those that occurred in ≥ 2 patients were decreased platelet count, decreased neutrophil count, constipation, and IRR (25.0%, each *n* = 2).

AEs were reported in all patients in the safety analysis population (Table 2). The most frequently reported AEs included CRS (75.0%, *n* = 6), aspartate aminotransferase increased (AST), alanine aminotransferase (ALT) increased and lymphocyte count decreased (50.0% each; *n* = 4). IRRs included grade 1 laryngeal pain and a grade 2 rash; both events occurred on the day of obinutuzumab administration and were resolved within the same day. The neutropenia observed in two patients who discontinued before glofitamab administration had not resolved when follow-up in the study was terminated.

**Table 2:** Adverse event summary

*n* (%)	Cohort 1 *n* = 5	Cohort 2 *n* = 3	All patients *N* = 8
At least one any grade AE	5 (100)	3 (100)	8 (100)
Glofitamab-related	3 (60.0)	3 (100)	6 (75.0)
Serious AEs	0	2 (66.7)	2 (25.0)
Glofitamab-related	0	2 (66.7)	2 (25.0)^a^
Grade 3–4 AEs	5 (100)	2 (66.7)	7 (87.5)
Glofitamab-related	3 (60.0)	2 (66.7)	5 (62.5)
Grade 5 (fatal) AE^b^	0	0	0
AE leading to discontinuation of glofitamab	2 (40.0)	2 (66.7)	4 (50.0)
Glofitamab-related	2 (40.0)	1 (33.3)	3 (37.5)^c^

^a^Glofitamab-related serious AEs were: COVID-19 infection (one patient) and jugular vein thrombosis and bacterial pneumonia (one patient); ^b^Not including PD; ^c^Glofitamab-related AEs leading to discontinuation were: COVID-19 infection (*n* = 2) and pneumonia (*n* = 1)

AE, adverse event; PD, progressive disease

Grade ≥ 3 AEs were reported in seven (87.5%) patients. Grade ≥ 3 AEs that occurred in at least two patients included decreased lymphocyte count (50.0%, *n* = 4), decreased platelet count, and neutropenia (25.0%, each *n* = 2). There were no grade 5 AEs. Serious AEs were reported in two (25.0%) patients and included COVID-19 infection (one patient), and jugular vein thrombosis and bacterial pneumonia (one patient). AEs leading to discontinuation of glofitamab occurred in four (50.0%) patients; these included COVID-19 infection (37.5%, *n* = 3) and pneumonia (12.5%, *n* = 1) and all were considered glofitamab-related except for COVID-19 infection in one patient. AEs leading to glofitamab interruption occurred in two (25.0%) patients: COVID-19 infection (*n* = 1), and jugular vein thrombosis and herpes zoster (*n* = 1). Glofitamab-related AEs were CRS (75.0%; *n* = 6), decreased lymphocyte count (50.0%; *n* = 4), neutropenia (37.5%; *n* = 3), and increased AST, increased ALT, COVID-19 infection, and anemia (25% each; *n* = 2).

Any grade CRS occurred in 75.0% of patients (*n* = 6; three in each cohort; grade 1, *n* = 3; grade 2, *n* = 2; grade 3, *n* = 1). In both cohorts, most patients experienced CRS during C1. CRS symptoms reported in at least two patients were pyrexia (75.0%, *n* = 6), hypotension, hypoxia, increased AST, and increased ALT (25.0% each, *n* = 2). One patient in Cohort 2 had recurrent fever with each glofitamab dose; all fever events were grade 1. Two patients with grade 2 and grade 3 CRS were administered oxygen. All six patients received steroids for CRS management. Two patients with grade 2 CRS (both in Cohort 2) received tocilizumab for CRS events which occurred on C1D8 and were resolved in both patients. The median duration of tocilizumab treatment was 1.5 days; no tocilizumab-related AEs were observed. No intensive care unit admission was required.

**Fig. 1:**
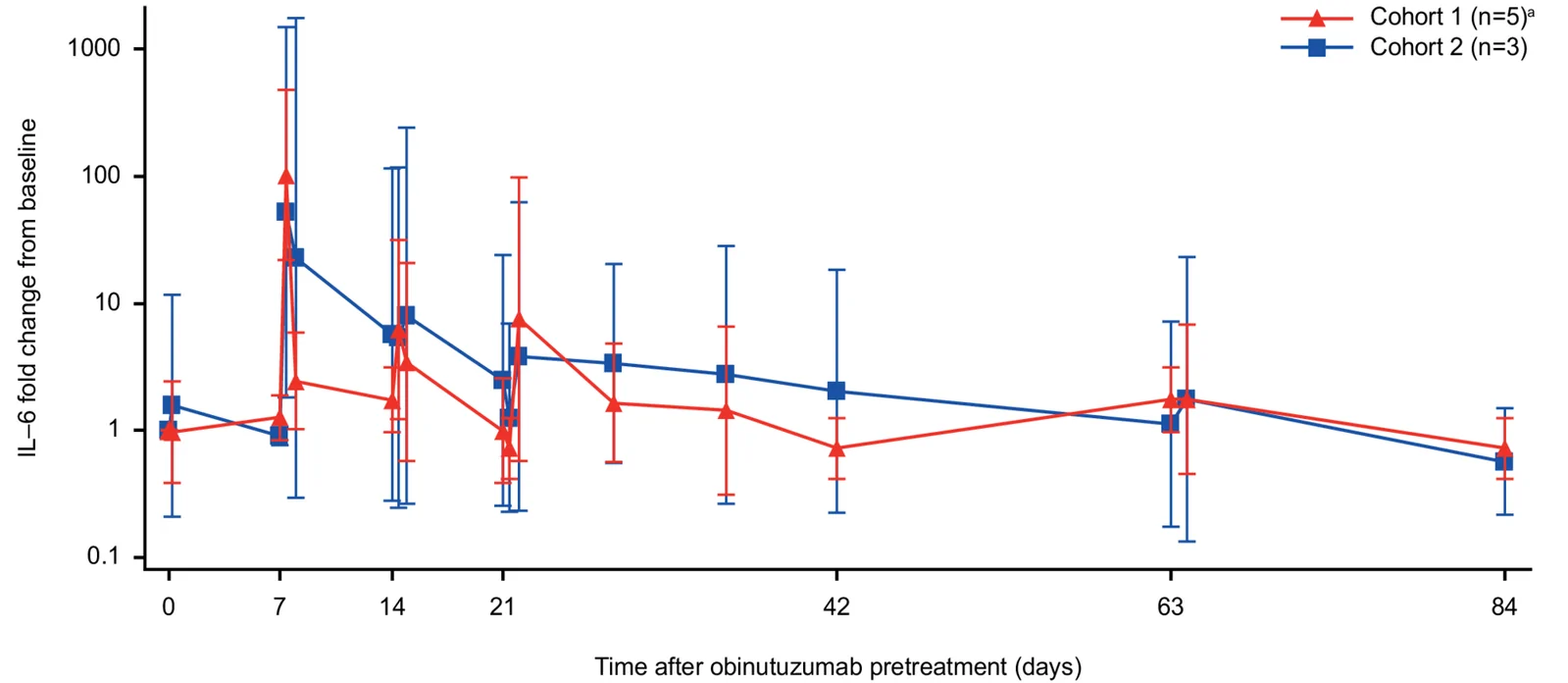
Levels of IL-6 post obinutuzumab and glofitamab administration in Cohort 1 (glofitamab 2.5/10/16 mg) and Cohort 2 glofitamab (2.5/10/30 mg). ^a^Two patients in Cohort 1 did not receive glofitamab. IL-6, interleukin-6

No IRRs were reported after glofitamab administration. An increase in IL-6 following glofitamab administration was primarily observed during the step-up dosing period, with lower levels of IL-6 elevation after step-up dosing (Fig. 1). No clinically significant changes in laboratory values were observed throughout the study.

### Pharmacokinetics

In Cohort 1 during C1–2 (first, second, and third dose), mean (SD) glofitamab C_max_ was 1.19 μg/mL (0.193) after the first dose, 5.98 μg/mL (1.23) after the second dose, and 6.70 μg/mL (2.49) after the third dose (Table 3). In Cohort 2 during C1–2 (first, second, and third dose), mean (SD) glofitamab C_max_ was 1.17 μg/mL (0.457) (first dose), 4.45 μg/mL (0.751) (second dose), and 13.5 μg/mL (3.05) (third dose). Mean (SD) AUC_0–35_ was 78.0 day*μg/mL (34.1) for Cohort 1 and 88.9 day*μg/mL (19.4) for Cohort 2.

**Table 3:** Pharmacokinetic analysis summary

PK parameter	Cohort 1 *n* = 5^a^	Cohort 2 *n* = 3
C_max_^b^, μg/mL, mean ± SD		
1st dose	1.19 ± 0.193	1.17 ± 0.457
2nd dose	5.98 ± 1.23	4.45 ± 0.751
3rd dose	6.70 ± 2.49	13.5 ± 3.05
AUC_0–35_, days*μg/mL, mean ± SD	78.0 ± 34.1	88.9 ± 19.4

^a^Two patients in Cohort 1 did not receive glofitamab. ^b^C_max_ in C1 and C2

AUC_0–35_, area under the concentration–time curve from time 0 to 35 days after the first glofitamab dose; C, cycle; C_max_, maximum serum concentration; PK, pharmacokinetic; SD, standard deviation

**Fig. 2:**
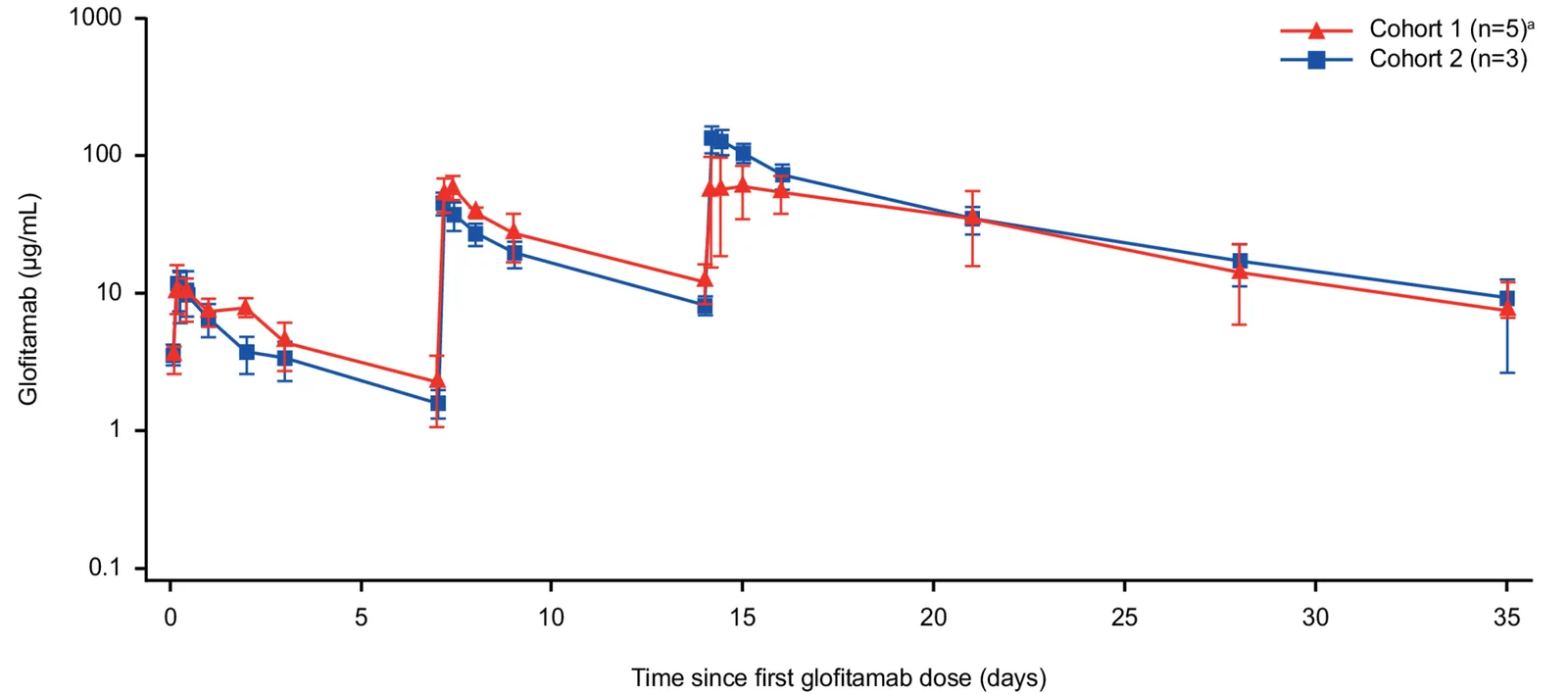
Mean serum concentration–time profiles for glofitamab following step-up dosing in Cohort 1 (glofitamab 2.5/10/16 mg) and Cohort 2 (2.5/10/30 mg). ^a^Two patients in Cohort 1 did not receive glofitamab and were excluded from pharmacokinetic analysis

The mean serum concentration–time profiles of glofitamab following step-up dosing showed similar time profiles across both cohorts, with exposure increasing in a dose-dependent manner (Fig. 2).

All patients tested negative for ADAs against glofitamab (Supplementary Appendix)**.**

### Efficacy

The ORR for all patients was 75.0% (*n* = 6/8; 95% CI 34.91–96.81) (Table 4). Efficacy assessments were not done for the two patients with FL who did not receive glofitamab. In Cohort 1, the ORR was 60.0% (*n* = 3/5; 95% CI 14.66–94.73) and all responses were CRs. The DOR ranged from 24.9 to 35.1 months and PFS ranged from 0.0 to 40.1 months. In Cohort 2, the ORR was 100% (*n* = 3/3; 95% CI 29.24–100.0); all three glofitamab-treated patients achieved a CR. The DOR ranged from 27.9 to 29.0 months and the PFS ranged from 30.0 to 31.8 months. All patients who received glofitamab in this study achieved a CR. Exploratory efficacy analysis results for glofitamab-treated patients can be found in the Supplementary Appendix.

**Table 4:** Efficacy analysis

n (%) [95% CI], unless otherwise specified	Cohort 1 *n* = 5^a^	Cohort 2 *n* = 3	All patients *N* = 8^a^
ORR	3 (60) [14.66–94.73]	3 (100) [29.24–100]	6 (75) [34.91–96.81]
CR	3 (60) [14.66–94.73]	3 (100) [29.24–100]	6 (75) [34.91–96.81]
PR	0 [0–52.18]	0 [0–70.76]	0 [0–36.94]
PD	0 [0–52.18]	0 [0–70.76]	0 [0–36.94]
DOR, months range	24.9–35.1	27.9–29.0	24.9–35.1
PFS, months range	0–40.1	30.0–31.8	0–40.1

^a^Includes two patients with follicular lymphoma who withdrew from the study prior to glofitamab administration; these patients did not undergo tumor assessment. Therefore, among the three patients who received glofitamab, three (100%) achieved a CR. CI, confidence interval; CR, complete response; DOR, duration of response; ORR, overall response rate; PD, disease progression; PFS, progression-free survival; PR, partial response

## Discussion

The anti-CD20xCD3 bispecific antibody glofitamab has previously demonstrated durable CRs and a manageable safety profile in R/R B-NHL [8, 9]. This study investigated the effects of glofitamab in Japanese patients with R/R B-NHL. Responses were durable, with 75.0% of patients achieving a CR. Glofitamab offered anti-tumor activity and a manageable safety profile, similar to those observed in the phase I/II trial of glofitamab monotherapy in a global population with R/R DLBCL after ≥ 2 prior therapies [8]. As no DLTs were observed thus the MTD was not reached, the recommended clinical dose was determined to be 2.5/10/30 mg based on the results of the pivotal phase I/II global study [8, 9]. No safety issues occurred in patients who received obinutuzumab pretreatment prior to glofitamab treatment. CRS was commonly reported but manageable. To mitigate high-grade CRS events, patients received obinutuzumab pretreatment and glofitamab was administered in step-up dosing, as done in previous studies [8, 15]. All CRS events were managed according to the protocol-specified guidelines and resolved with either corticosteroids or corticosteroids combined with tocilizumab, depending on the severity of the event.

In this study, unlike in the pivotal phase I/II study, glofitamab was administered until PD rather than as a fixed-duration treatment. Based on the mechanism of action of glofitamab, prolonged treatment may result in sustained B-cell depletion and subsequent hypogammaglobulinemia which could be associated with an increased risk of infections. Of note, all four AEs leading to glofitamab discontinuation in this study were infections, comprising three cases of COVID-19 infection and one case of pneumonia. Furthermore, two patients discontinued treatment due to hematological toxicities following obinutuzumab pretreatment rather than glofitamab itself. While one of these patients had bone marrow involvement, both patients had received four or more prior lines of therapy, which likely exacerbated their baseline myelosuppression. This suggests that when bone marrow infiltration or myelosuppression from prior therapy is observed, appropriate management such as prophylactic administration (e.g. granulocyte-colony stimulating factor) and careful monitoring is required to mitigate the risk of severe hematological toxicities.

This is the first study to evaluate glofitamab monotherapy specifically in Japanese patients. Whilst tumor activity and the safety profile were as expected based on previous studies [8, 15], there was a difference in the glofitamab PK profile; glofitamab exposures in Japanese patients during C1 and C2 were slightly higher than those observed in the global study population [9]. This may be attributable to differences in baseline patient characteristics; for example, the patients included in this study typically had a lower body weight compared with those in the global study [8, 9].

No antibodies to glofitamab were detected in any patient during the course of the study. Although IL-6 elevation was primarily observed during step-up dosing of glofitamab, this is consistent with the data showing that CRS rarely occurred post step-up dosing [8, 9].

The ORR with glofitamab monotherapy was 75.0%, and all six glofitamab-treated patients achieved a CR. The efficacy of glofitamab in Japanese patients with DLBCL (and one patient with trFL) in this study, is consistent with the efficacy data reported in the global phase I/II studies evaluating glofitamab in R/R B-NHL (ORR 66%; CR 57% at the recommended phase II dose [RP2D; 2.5/10/30 mg]) and R/R DLBCL (ORR 52%; CR 39%) [8, 9]. The response rates are also comparable with those achieved with available treatment options for R/R large B-cell lymphoma (LBCL) in global studies in the second-line setting and beyond, including CAR T-cell therapy in transplant-eligible and transplant-ineligible patients (ORR 50–80%) [16–23], and combination therapies such as tafasitamab-lenalidomide (ORR 60%; CR 43%) [24] and polatuzumab vedotin plus bendamustine and rituximab (ORR 53%; CR 34%) [25]. ORR and CR rates reported in studies of other CD20xCD3 antibodies as monotherapy for R/R DLBCL are 63% and 40% for epcoritamab [26] and 52% and 32% for odronextamab [27], respectively. Of course, caution is needed when comparing across studies due to the differences in disease characteristics, patient populations, and the variability due to the small sample size herein.

Based on the clinical activity of glofitamab monotherapy observed here and the encouraging clinical activity observed in the global phase I/II trial [8, 9], it can be expected that glofitamab monotherapy may have similar efficacy and a manageable safety profile in Japanese patients with R/R B-NHL. These preliminary efficacy findings suggest that glofitamab monotherapy demonstrates promising activity in Japanese patients with R/R NHL. Glofitamab monotherapy could become a reasonable option for this population in the future.

A limitation of the study was its small sample size (*n* = 8); Furthermore, although patients with various NHL subtypes, including DLBCL, FL and trFL, were eligible for inclusion in the study, no patients with FL were ultimately treated with glofitamab. Consequently, the efficacy of glofitamab specifically in patients with FL could not be evaluated in this study. It should be noted that the enrolled patient population herein consists of Japanese patients selected according to the trial’s eligibility criteria and may not be fully representative of patients encountered in routine clinical practice.

In this trial, the safety profile of glofitamab monotherapy was tolerable and manageable in Japanese patients with R/R B-NHL and similar to the safety profile seen in global populations [8, 9]. No DLTs were observed. Glofitamab achieved encouraging response rates, which were durable, and glofitamab exposure in Japanese patients was slightly higher than that reported in the global phase I/II study. In conclusion, glofitamab monotherapy indicated a manageable safety profile with promising anti-tumor activity in Japanese patients with R/R B-NHL. These results contribute to the growing body of evidence supporting glofitamab in R/R B-NHL.

## Supplementary Information

Below is the link to the electronic supplementary material.

Supplementary Material 1

## Data Availability

Qualified researchers may request access to individual patient-level data through the clinical study data request platform (https://www.clinicalstudydatarequest.com. For further details on Chugai’s Data Sharing Policy and how to request access to related clinical study documents, see https://www.chugai-pharm.co.jp/english/profile/rd/ctds_request.html.
