# Supplementary material for: Phase I study of glofitamab in Japanese patients with relapsed or refractory B-cell non-Hodgkin lymphoma
Source: Int J Clin Oncol. 2026 Jul 2;31(9):1975–83. doi: 10.1007/s10147-026-03110-2 (PMC13506615; doi:10.1007/s10147-026-03110-2)
Supplement: Supplementary file 1 [file 10147_2026_3110_MOESM1_ESM.docx]

**Supplementary Appendix**

Phase I study of glofitamab in Japanese patients with relapsed or refractory
B-cell non-Hodgkin lymphoma

**Target journal:** International Journal of Clinical Oncology

**Authors:** Yuko Shirouchi^1^*, Yuko Mishima^1^, Suguru Fukuhara^2^, Shinichi Makita^2^,
Koji Izutsu^2^, Yasuhito Terui^3^, Takuro Suzuki^4^, Atsuko Kawasaki^4^, Takeshi Miyake^4^, Dai Maruyama^1^

**Affiliations:** ^1^Department of Hematology Oncology, Cancer Institute Hospital, Japanese Foundation for Cancer Research, Tokyo, Japan; ^2^Department of Hematology, National Cancer Center Hospital, Tokyo, Japan; ^3^Department of Hematology, Saitama Medical University Hospital, Saitama, Japan; ^4^Chugai Pharmaceutical Co., Ltd, Tokyo, Japan

***Author for correspondence:** Yuko Shirouchi; email: yuko.shirouchi@jfcr.or.jp; phone: +81 3 3520 0111, ORCID number: 0000-0003-0945-2030

**Methods**

*Study design*

The doses and schedules were selected based on the previously published safety and efficacy data obtained from the prior global phase I/II trial of glofitamab [1]. During the global phase I/II trial it was established that step-up dosing and obinutuzumab pretreatment can mitigate the risk of cytokine release syndrome (CRS) and increase glofitamab efficacy.

*CRS management*

Management for CRS depends on the severity of the event. If grade 1 CRS occurs during glofitamab administration, treatment should be interrupted until the event has resolved itself. If CRS persists for more than two days, tocilizumab is recommended. The addition of corticosteroids to tocilizumab may be considered at the discretion of the investigator or sub investigator. For grade 2 CRS, glofitamab should be discontinued until the next scheduled dosing date and tocilizumab and corticosteroids should be administered. If there is no improvement 8–12 hours after tocilizumab initiation, management in the intensive care unit (ICU) should be considered. If grade 3 CRS occurs, patients should be premedicated with acetaminophen and an antihistamine for all subsequent glofitamab infusions. Corticosteroids and tocilizumab should be administered and hospitalization considered for management and next dose date. If grade 3 or higher CRS recurs, glofitamab should be discontinued permanently. If grade 4 CRS occurs, glofitamab should be discontinued; tocilizumab and corticosteroids should be administered, and the patient should be managed in the ICU.

*Inclusion criteria*

Patients who met the following criteria were included in the study:

- Have received a full explanation of the details of the study and given their written consent.
- Age at the time of informed consent 20 years or older.
- Eastern Cooperative Oncology Group performance status (ECOG PS) of 0 or 1.
- Patients are expected to survive for at least 12 weeks from the date of enrollment.
- Histologically confirmed CD20-positive B-cell non-Hodgkin lymphoma (B-NHL) including any of the following: follicular lymphoma (FL), diffuse large B-cell lymphoma (DLBCL), marginal zone lymphoma, mantle cell lymphoma, primary mediastinal large B-cell lymphoma, or transformed FL.
- For relapsed or refractory (R/R) B-NHL, there is no alternative preferred standard treatment (standard chemotherapy, autologous hematopoietic stem cell transplantation, etc.).
- Able to provide tumor tissue samples that meet all of the following criteria:
  - Tumor tissue samples treated with formalin-fixed paraffin block or unstained slides
  - Samples collected after the last dose of prior treatment (however, even if this procedure cannot be performed, substitution with a sample before the last dose of the previous treatment may be accepted after prior discussion with the sponsor)
  - There should be no findings suggestive of transformation or histological type change from the time of tumor tissue sample collection.
- Before enrollment major organ function within two weeks meets all of the following criteria. If more than one test result is available during this period, the latest one before enrollment will be adopted.
  - Neutrophil count 1,500/μL or more
  - Platelet count 75,000/μL or more
  - Hemoglobin 9.0 g/dL or higher
  - Aspartate aminotransferase (AST) and/or alanine aminotransferase (ALT) ≤3x upper limit of normal (ULN)
  - Total bilirubin	≤1.5x ULN (if Gilbert's syndrome, ≥3x ULN)
  - Serum creatinine ≤1.5x ULN or creatinine clearance (Clearance, CL) 50mL/min more than that (creatinine CL will be calculated either by actual measurement or by the Cockcroft-Gault formula).
    Cockcroft-Gault formula
    - Male: [(140 minus age) x weight (kg)] / [72 x serum creatinine (mg/dL)]
    - Female: [(140 minus age) x weight (kg) x 0.85] / [72 x serum creatinine (mg/dL)]
- Prior treatment or procedure must have been completed at least the following durations prior to the planned date of administration of obinutuzumab:
  - Surgical procedures: four weeks
    - Major surgery requiring thoracotomy/laparotomy or laparoscopic organ resection. However, if a biopsy specified in the protocol (tumor biopsy, bone marrow biopsy) has been performed within the above period, the patient may be enrolled if the event has resolved to the point where the investigator or sub-investigator can judge that there is no bleeding risk at the time of enrollment.
  - Chemotherapy: four weeks
    - Chemotherapy includes molecularly targeted drugs.
  - Antibody therapy: four weeks
    - Antibody therapy includes monoclonal antibodies, radioimmunoconjugates and antibody-drug conjugates.
  - Immunotherapy in oncology: four weeks
    - Including immune checkpoint inhibitors (anti-programmed cell death protein 1 / programmed cell death ligand 1 antibody drugs, anti-cytotoxic T lymphocyte-associated antigen 4 antibody drugs, etc.) and co-stimulatory agonists (anti-CD 137 antibody drugs, anti-CD 27 antibody drugs, anti-glucocorticoid-induced tumor necrosis factor [TNF] receptor-associated protein antibody drugs, anti-CD 40 antibody drugs, etc.), and all other cancer immunotherapy (immunomodulators such as interferon [IFN]-α, IFN-y, interleukin [IL]-2, bioresponse modulator therapy, cancer vaccine therapy, oncology specific drugs including tumor-specific T-cell infusion therapy)
  - Radiotherapy: four weeks
    - After completion of palliative radiotherapy for pain relief, enrollment is allowed if at least two weeks have passed
  - Autologous stem cell transplant: 12 weeks
  - Transfusions: two weeks
  - Platelet transfusion: two weeks
  - Immunosuppressive therapy, two weeks
    - Prednisolone, cyclophosphamide, azathioprine, methotrexate, thalidomide, TNF-α and antagonists are permitted. Topical, inhaled corticosteroids, or 25mg/Day prednisolone or equivalent doses are permitted.
  - Live vaccine and attenuated vaccine: four weeks
  - Other investigational product: four weeks

*Exclusion criteria*

The presence of any of the following will exclude a patient from enrollment:

- The patient has a history of hypersensitivity to the excipients of obinutuzumab (histidine, trehalose, poloxamer 188), and the excipients of glofitamab (sucrose, methionine, and polysorbate 20).
- Known history of severe allergic or anaphylactic reactions to monoclonal antibody therapy (chimeric antibodies, humanized antibodies, and human antibodies) or fusion proteins.
- Has a current or past history of central nervous system lymphoma, metastases to the central nervous system, or metastases to meninges.
- The patient had multiple cancers with a disease-free interval of less than two years. However, malignancies that are considered unlikely to recur, such as completely resected basal cell carcinoma of the skin and cervical intraepithelial neoplasia, gastrointestinal cancers that have been completely resected by endoscopic mucosal resection, or malignancies that do not require treatment, such as early prostate cancer, are excluded.
- A serious adverse event related to obinutuzumab that was associated with a previous dose of obinutuzumab was reported, which led to discontinuation of the obinutuzumab.
- Persistent adverse reactions associated with prior treatment of grade 2 or higher as defined in the NCI CTCAE (v 5.0). However, alopecia and lymphocyte count decreased will be permitted.
- History or presence of any of the following adverse reactions to prior immunotherapy (excluding endocrinopathies manageable with hormone replacement therapy):
  - NCI CTCAE (v5.0) grade 3 or higher adverse reactions
  - Adverse reactions of NCI CTCAE (v5.0) grade 1, grade 2 not expected to resolve by the time of enrollment in this study.
- Active infection (includes bacterial, viral, fungal, mycobacterial, Epstein-Barr virus (EBV), hepatitis B, hepatitis C, human immunodeficiency virus (HIV), tuberculosis, and other infections. Exclude fungal infection of nail bed within 4 weeks prior to dosing with obinutuzumab in this study.
- Prior to the administration of obinutuzumab in this study presence (including history) of infection requiring intravenous antibiotic therapy or hospitalization within four weeks. A positive result for EBV, cytomegalovirus (CMV), human T-lymphotropic virus type 1 precludes enrollment, even if the infection is inactive.
- Presence or history of autoimmune disease. However, the following cases are allowed:
  - Presence or history of hypothyroidism that is controlled with stable thyroid hormone replacement therapy
  - Presence or history of immune thrombocytopenic purpura or autoimmune hemolytic anemia
  - Controllable concurrent or previous type 1 diabetes mellitus (HbA1c <8% and no ketoacidosis at screening)
- Prior allogeneic hematopoietic stem cell transplantation or organ transplantation.
- HIV, positive for antibodies.
- Hepatitis B surface (HBs) antigen positive. However, patients who are negative for HBs antigen and show only positive for HBs antibody and have a clear history of vaccination for hepatitis B virus (HBV), and patients who are negative for HBs antigen and show positive for HBs antibody and/or positive for Hepatitis B core (HBc) antibody and do not have detectable HBV-DNA are eligible for enrollment.
- Hepatitis C virus (HCV), positive for antibody. However, patients who are positive for HCV antibody and negative for HCV-RNA can be enrolled.
- The patient had a history or complication of progressive multifocal leukoencephalopathy (PML).
- Presence or history of macrophage activation syndrome/hemophagocytic lymphohistiocytosis.
- History or presence of significant pulmonary disease, including severe obstructive pulmonary disease.
- Have New York Heart Association Class III or greater cardiac disease, arrhythmia, or unstable angina, and a history or complication of myocardial infarction within six months before enrollment.
- Patients with current or previous central nervous system disease (stroke, epilepsy, central nervous system vasculitis, neurodegenerative disease, etc.). However, patients with a history of stroke, may be enrolled if the investigator or sub-investigator determines that they have not had a stroke or transient ischemic attack in the past two years and have no residual neurological deficits.
- Clinically significant hepatic disease (viral, alcoholic or other hepatitis, hepatic cirrhosis, etc.).
- Uncontrollable hypertension (systolic blood pressure ≥140mmHg or diastolic blood pressure ≥90mmHg).
- Uncontrolled diabetes mellitus.
- Pregnant or lactating women (lactating females can be enrolled if they agree to discontinue breastfeeding for the duration of the study and for 18 months after the last dose of obinutuzumab, two months after the last dose of glofitamab, or three months after the last dose of tocilizumab, whichever is longer).
- Women with a positive pregnancy test (a woman who has not been in a postmenopausal state [≥12 months of amenorrhea without an identified cause other than menopause] and has not undergone an oophorectomy and/or hysterectomy is considered a woman of childbearing potential and will undergo pregnancy testing. However, even if the patient has no menstruation within 12 months, a pregnancy test will be performed in women for whom the possibility of pregnancy cannot be ruled out for reasons such as chemical menopause).
- A female of childbearing potential who is not willing to use adequate contraception or not donate eggs during the study and for 18 months after the last dose of obinutuzumab, two months after the last dose of glofitamab, or three months after the last dose of tocilizumab, whichever is longer.
- A male who is not willing to use adequate contraception or not donate sperm for the duration of the study and for three months after receiving obinutuzumab, two months after the last dose of glofitamab, or two months after the last dose of tocilizumab, whichever is longer.
- Previously treated with a bispecific anti-CD20 antibody targeting both CD20 and CD3.
- The investigator or sub-investigator judges that participation in this study is inappropriate for any other reason.

*Treatment discontinuation criteria*

Decisions on treatment discontinuation were made using thorough benefit-risk evaluation based on the most up to date clinical trial information available.

The following events are considered sufficient reasons for discontinuing a patient from the investigational product: re-enrollment in this study will not be allowed.

- The patient becomes pregnant.
- The patient has progressive disease (PD). Even if the PD is assessed according to the Lugano Response Criteria (with modification), the continuation of treatment will be permitted as long as all of the following criteria are met and the investigator or sub-investigator judges it appropriate to continue the treatment with glofitamab after considering the benefits and risks for the patient:
  - No clinical symptoms or signs indicating PD
  - No worsening of ECOG PS from baseline
- If dose-limiting toxicity (DLT) occurs during the DLT evaluation period. However, even if DLT occurs, if the event occurs early in the treatment phase for which a causal relationship is recognized only to glofitamab (CRS, tumor flare, etc.) and the event resolves/improves to grade 1 or lower, the continuation of administration at a dose not exceeding the specified dose of glofitamab on the next scheduled day will be permitted after discussion with the sponsor if the investigator or sub-investigator judges it appropriate to continue administration of glofitamab after considering the benefits/risks for patients. If the study treatment is to be continued, the patient should be fully informed of the potential risks associated with continued study treatment and written consent to continue study treatment should be obtained from the patient.
- If any adverse event corresponding to discontinuation as specified is observed.
- The patient experiences an adverse event (including aggravation of a concurrent medical condition) that makes it difficult for them to continue the study.
- Administration of antineoplastic therapy other than the study treatment.
- The patient wishes to discontinue the study drug.
- Significant violation of eligibility criteria was found after enrollment.
- The Cycle (C)1 Day (D)1 dosing criteria for glofitamab are not met.
- The dose of glofitamab is missed for more than six weeks (two weeks for C1D8, C2D1, four weeks for C3D1) after the last dose due to reasons such as patient's convenience or adverse events.
- The investigator or sub-investigator judges that continuation of the study is inappropriate for any other reason.

*Pharmacokinetic (PK) analysis population*

Patients who received one dose of glofitamab and had at least one serum glofitamab concentration measurement or received at least one dose of obinutuzumab and had at least one serum obinutuzumab concentration measurement were included in the PK analysis.

*Definitions of secondary efficacy endpoints*

Investigator-assessed ORR, defined as the proportion of responders in the overall population.

Duration of response [DOR], defined as the time from the earliest occurrence of CR or partial response [PR] to the date of PD or death.

Progression-free survival [PFS], defined as the time from the study start date to the earliest occurrence of PD or death, whichever occurred first.

**Supplementary Results**

*Exploratory efficacy analysis of glofitamab-treated patients*

In the efficacy analysis of glofitamab-treated patients (Cohort 1, n=3; Cohort 2, n=3), the CR rate was 100% (95% CI: 29.24–100.0) in Cohort 1, and 100% (95% CI: 54.07–100.0) across both cohorts. PFS ranged from 27.2–40.1 months (Cohorts 1 and 2) and DOR was 24.9–35.1 months (Cohorts 1 and 2).

*Immunogenicity*

Anti-drug antibodies (ADAs) were evaluated in all patients (n=8) before obinutuzumab treatment and in six patients after obinutuzumab treatment. Samples for ADA assessment were collected every cycle up to C8 and every eight cycles thereafter. No patients tested positive for antibody to glofitamab at any time before or after administration of obinutuzumab.

**Reference**

1. Hutchings M, Morschhauser F, Iacoboni G, Carlo-Stella C, Offner FC, Sureda A, Salles G, Martínez-Lopez J, Crump M, Thomas DN, Morcos PN, Ferlini C, Bröske AE, Belousov A, Bacac M, Dimier N, Carlile DJ, Lundberg L, Perez-Callejo D, Umaña P, Moore T, Weisser M, Dickinson MJ (2021) Glofitamab, a novel, bivalent CD20-targeting T-cell-engaging bispecific antibody, induces durable complete remissions in relapsed or refractory B-cell lymphoma: a phase I trial. J Clin Oncol 39 (18):1959-1970. doi:10.1200/jco.20.03175
